# Supplementary material for: Structural basis for mTORC1 regulation by the CASTOR1–GATOR2 complex
Source: Nat Struct Mol Biol. 2025 Jul 25;32(10):1980–8. doi: 10.1038/s41594-025-01635-0 (PMC12477443; doi:10.1038/s41594-025-01635-0)
Supplement: Supplementary file 1 — Reporting Summary [file 41594_2025_1635_MOESM1_ESM.pdf]

nature portfolio

Corresponding author(s): James H. Hurley

Last updated by author(s): Jun. 10, 2025

Reporting Summary

Nature Portfolio wishes to improve the reproducibility of the work that we publish. This form provides structure for consistency and transparency in reporting. For further information on Nature Portfolio policies, see our Editorial Policies and the Editorial Policy Checklist.

Statistics

For all statistical analyses, confirm that the following items are present in the figure legend, table legend, main text, or Methods section.

n/a	Confirmed	
☐	☒	The exact sample size (*n*) for each experimental group/condition, given as a discrete number and unit of measurement
☒	☐	A statement on whether measurements were taken from distinct samples or whether the same sample was measured repeatedly
☒	☐	The statistical test(s) used AND whether they are one- or two-sided<br>*Only common tests should be described solely by name; describe more complex techniques in the Methods section.*
☒	☐	A description of all covariates tested
☒	☐	A description of any assumptions or corrections, such as tests of normality and adjustment for multiple comparisons
☐	☒	A full description of the statistical parameters including central tendency (e.g. means) or other basic estimates (e.g. regression coefficient) AND variation (e.g. standard deviation) or associated estimates of uncertainty (e.g. confidence intervals)
☒	☐	For null hypothesis testing, the test statistic (e.g. *F*, *t*, *r*) with confidence intervals, effect sizes, degrees of freedom and *P* value noted<br>*Give P values as exact values whenever suitable.*
☒	☐	For Bayesian analysis, information on the choice of priors and Markov chain Monte Carlo settings
☒	☐	For hierarchical and complex designs, identification of the appropriate level for tests and full reporting of outcomes
☒	☐	Estimates of effect sizes (e.g. Cohen's *d*, Pearson's *r*), indicating how they were calculated

Our web collection on statistics for biologists contains articles on many of the points above.

Software and code

Policy information about availability of computer code

Data collection	SerialEM, CryoSPARC v4.3.1 and v4.4.1
Data analysis	Chimera X, COOT 0.9.8. Statistical analysis was performed using GraphPad Prism Version 10.2.0 (335).

For manuscripts utilizing custom algorithms or software that are central to the research but not yet described in published literature, software must be made available to editors and reviewers. We strongly encourage code deposition in a community repository (e.g. GitHub). See the Nature Portfolio guidelines for submitting code & software for further information.

Data

Policy information about availability of data

All manuscripts must include a data availability statement. This statement should provide the following information, where applicable:

- Accession codes, unique identifiers, or web links for publicly available datasets
- A description of any restrictions on data availability
- For clinical datasets or third party data, please ensure that the statement adheres to our policy

The coordinates and density map have been deposited in the RCSB and EMDB with accession codes 9OTI and EMD-70833. Focused maps have been deposited in the EMDB with accession codes EMD-71136, EMD-71137, EMD-71138, EMD-71139, EMD-71140, EMD-71141, and EMD-71143.

Field-specific reporting

Please select the one below that is the best fit for your research. If you are not sure, read the appropriate sections before making your selection.

☒ Life sciences ☐ Behavioural & social sciences ☐ Ecological, evolutionary & environmental sciences

For a reference copy of the document with all sections, see nature.com/documents/nr-reporting-summary-flat.pdf

Life sciences study design

All studies must disclose on these points even when the disclosure is negative.

Sample size	Sample sizes were not predetermined using statistical methods. The use of three replicates is customary in our field.
Data exclusions	No data were excluded from analysis
Replication	All experiments were successfully replicated three times, except for the extended figure 9f, which was replicated two times.
Randomization	Randomization is not considered customary or necessary in this field.
Blinding	Blinding is not considered customary or necessary in this field.

Reporting for specific materials, systems and methods

We require information from authors about some types of materials, experimental systems and methods used in many studies. Here, indicate whether each material, system or method listed is relevant to your study. If you are not sure if a list item applies to your research, read the appropriate section before selecting a response.

Materials & experimental systems

n/a	Involved in the study	
☐	☒	Antibodies
☐	☒	Eukaryotic cell lines
☒	☐	Palaeontology and archaeology
☒	☐	Animals and other organisms
☒	☐	Human research participants
☒	☐	Clinical data
☒	☐	Dual use research of concern

Methods

n/a	Involved in the study	
☒	☐	ChIP-seq
☒	☐	Flow cytometry
☒	☐	MRI-based neuroimaging

Antibodies

Antibodies used

MIOS (Cell Signaling Technology, Cat#13557S, Clone#D12C6, Lot#1), WDR59 (Cell Signaling Technology, Cat#53385S, Clone#D4Z7A, Lot#1), FLAG (Cell Signaling Technology, Cat#14793S, Clone#D6W5B, Lot#7), HA (Cell Signaling Technology, Cat#3724S, Clone#C29F4, Lot#11), S6K1 (Cell Signaling Technology, Cat#2708S, Clone#49D7, Lot#8), phospho-T389-S6K1 (Cell Signaling Technology, Cat#9234S, Clone#108D2, Lot#16). Antibodies were used at the following dilutions: MIOS 1:1000 (Cell Signaling Technology, Cat#13557S, Clone#D12C6, Lot#1), WDR59 1:1000 (Cell Signaling Technology, Cat#53385S, Clone#D4Z7A, Lot#1), FLAG 1:1000 (Cell Signaling Technology, Cat#14793S, Clone#D6W5B, Lot#7), HA 1:1000 (Cell Signaling Technology, Cat#3724S, Clone#C29F4, Lot#11), S6K1 1:1000 (Cell Signaling Technology, Cat#2708S, Clone#49D7, Lot#8), phospho-T389-S6K1 1:1000 (Cell Signaling Technology, Cat#9234S, Clone#108D2, Lot#16).

Validation

MIOS (Cell Signaling Technology, Cat#13557S, Clone#D12C6, Lot#1), monoclonal antibody produced by immunizing animals with a synthetic peptide corresponding to residues surrounding Leu730 of human Mios protein. Recognizes endogenous levels of total Mios protein from Human, Mouse and Rat by Western blot (https://www.cellsignal.com/products/primary-antibodies/mios-d12c6-rabbit-mab/13557?srsltid=AfmBOopX3nLp8fdT5IsdpECXzToUYElh7UA95rwj0wb2zOec0FZb8gT8).

WDR59 (Cell Signaling Technology, Cat#53385S, Clone#D4Z7A, Lot#1), monoclonal antibody produced by immunizing animals with a synthetic peptide corresponding to residues surrounding His356 of human WDR59 protein. Recognizes endogenous levels of total WDR59 protein from Human and Monkey by Western blot (https://www.cellsignal.com/products/primary-antibodies/wdr59-d4z7a-rabbit-mab/53385?srsltid=AfmBOoqJ0MRPgVUUZ8vXJJyriFfm-EUD_Fn11MA0R5k0Pu_c-EZOcN8F).

FLAG (Cell Signaling Technology, Cat#14793S, Clone#D6W5B, Lot#7), monoclonal antibody produced by immunizing animals with a synthetic DYKDDDDK peptide. Detects exogenously expressed DYKDDDDK proteins in cells. By Western blot, the antibody recognizes the DYKDDDDK peptide, which is the same epitope recognized by Sigma-Aldrich Anti-FLAG M2 antibody, fused to either the amino-terminus or carboxy-terminus of the target protein (https://www.cellsignal.com/products/primary-antibodies/dykddddk-tag-d6w5b-rabbit-mab-binds-to-same-epitope-as-sigma-aldrich-anti-flag-m2-antibody/14793?srsltid=AfmBOor5TWqJlYICgfOXmddb9d7FC8x5rEF_EGz8sAKmj7yZlczklp-c).

HA (Cell Signaling Technology, Cat#3724S, Clone#C29F4, Lot#11), monoclonal antibody produced by immunizing animals with a synthetic peptide containing the influenza hemagglutinin epitope (YPYDVPDYA). By western blot, detects exogenously expressed proteins containing the HA epitope tag. The antibody may cross-react with a protein of unknown origin ~100kDa (https://www.cellsignal.com/products/primary-antibodies/ha-tag-c29f4-rabbit-mab/3724?srsltid=AfmBOooWThRdxyoWIsodZBfJjx5jJ_Me--yDubOnEwlcJ8pLYc8F7kum).

S6K1 (Cell Signaling Technology, Cat#2708S, Clone#49D7, Lot#8), monoclonal antibody produced by immunizing animals with a synthetic peptide corresponding to residues surrounding the amino-terminus of human p70 S6 kinase. Detects endogenous levels of total p70 S6 kinase protein from Human by Western blot. The antibody also recognizes p85 S6 kinase (https://www.cellsignal.com/products/primary-antibodies/p70-s6-kinase-49d7-rabbit-mab/2708?srsltid=AfmBOoqKAFLvrXPGZDqJ0MH1Abfbij7FXPpaD09AdQP2YqXg-grA4fhp)

phospho-T389-S6K1 (Cell Signaling Technology, Cat#9234S, Clone#108D2, Lot#16), monoclonal antibody produced by immunizing animals with a synthetic phosphopeptide corresponding to residues surrounding Thr389 of human p70 S6 kinase. Detects endogenous levels of p70 S6 kinase only when phosphorylated at Thr389 from Human, Mouse, Rat and Mondey by Western blot. This antibody also detects p85 S6 kinase when phosphorylated at the analogous site (Thr412) and possibly S6KII phosphorylated at Thr388. This antibody may detect a non-specific band that runs around 62 kDa in some samples. The band is not phosphatase sensitive (https://www.cellsignal.com/products/primary-antibodies/phospho-p70-s6-kinase-thr389-108d2-rabbit-mab/9234?srsltid=AfmBOorVPkWzz4ryrCic7_SExN4cJbn2_8QaY6ydTEz5oo_b-omfCT2G)

MIOS (Cell Signaling Technology, Cat#13557S, Clone#D12C6, Lot#1), monoclonal antibody produced by immunizing animals with a synthetic peptide corresponding to residues surrounding Leu730 of human Mios protein. Recognizes endogenous levels of total Mios protein from Human, Mouse and Rat by Western blot (https://www.cellsignal.com/products/primary-antibodies/mios-d12c6-rabbit-mab/13557?srsltid=AfmBOopX3nLp8fdT5IsdpECXzToUYElh7UA95rwj0wb2zOec0FZb8gT8).

WDR59 (Cell Signaling Technology, Cat#53385S, Clone#D4Z7A, Lot#1), monoclonal antibody produced by immunizing animals with a synthetic peptide corresponding to residues surrounding His356 of human WDR59 protein. Recognizes endogenous levels of total WDR59 protein from Human and Monkey by Western blot (https://www.cellsignal.com/products/primary-antibodies/wdr59-d4z7a-rabbit-mab/53385?srsltid=AfmBOoqJ0MRPgVUUZ8vXJJyriFfm-EUD_Fn11MA0R5k0Pu_c-EZOcN8F).

FLAG (Cell Signaling Technology, Cat#14793S, Clone#D6W5B, Lot#7), monoclonal antibody produced by immunizing animals with a synthetic DYKDDDDK peptide. Detects exogenously expressed DYKDDDDK proteins in cells. By Western blot, the antibody recognizes the DYKDDDDK peptide, which is the same epitope recognized by Sigma-Aldrich Anti-FLAG M2 antibody, fused to either the amino-terminus or carboxy-terminus of the target protein (https://www.cellsignal.com/products/primary-antibodies/dykddddk-tag-d6w5b-rabbit-mab-binds-to-same-epitope-as-sigma-aldrich-anti-flag-m2-antibody/14793?srsltid=AfmBOor5TWqJlYICgfOXmddb9d7FC8x5rEF_EGz8sAKmj7yZlczklp-c).

HA (Cell Signaling Technology, Cat#3724S, Clone#C29F4, Lot#11), monoclonal antibody produced by immunizing animals with a synthetic peptide containing the influenza hemagglutinin epitope (YPYDVPDYA). By western blot, detects exogenously expressed proteins containing the HA epitope tag. The antibody may cross-react with a protein of unknown origin ~100kDa (https://www.cellsignal.com/products/primary-antibodies/ha-tag-c29f4-rabbit-mab/3724?srsltid=AfmBOooWThRdxyoWIsodZBfJjx5jJ_Me--yDubOnEwlcJ8pLYc8F7kum).

S6K1 (Cell Signaling Technology, Cat#2708S, Clone#49D7, Lot#8), monoclonal antibody produced by immunizing animals with a synthetic peptide corresponding to residues surrounding the amino-terminus of human p70 S6 kinase. Detects endogenous levels of total p70 S6 kinase protein from Human by Western blot. The antibody also recognizes p85 S6 kinase (https://www.cellsignal.com/products/primary-antibodies/p70-s6-kinase-49d7-rabbit-mab/2708?srsltid=AfmBOoqKAFLvrXPGZDqJ0MH1Abfbij7FXPpaD09AdQP2YqXg-grA4fhp)

phospho-T389-S6K1 (Cell Signaling Technology, Cat#9234S, Clone#108D2, Lot#16), monoclonal antibody produced by immunizing animals with a synthetic phosphopeptide corresponding to residues surrounding Thr389 of human p70 S6 kinase. Detects endogenous levels of p70 S6 kinase only when phosphorylated at Thr389 from Human, Mouse, Rat and Mondey by Western blot. This antibody also detects p85 S6 kinase when phosphorylated at the analogous site (Thr412) and possibly S6KII phosphorylated at Thr388. This antibody may detect a non-specific band that runs around 62 kDa in some samples. The band is not phosphatase sensitive (https://www.cellsignal.com/products/primary-antibodies/phospho-p70-s6-kinase-thr389-108d2-rabbit-mab/9234?srsltid=AfmBOorVPkWzz4ryrCic7_SExN4cJbn2_8QaY6ydTEz5oo_b-omfCT2G)

Eukaryotic cell lines

Policy information about cell lines

Cell line source(s)	Human Embryonic Kidney cells (HEK-293T) were obtained from ATCC (https://www.atcc.org/).
Authentication	HEK-293T was verified by ATCC (https://www.atcc.org/products/crl-3216), as well as by morphological analysis.
Mycoplasma contamination	All cel lines used in this study were tested negative for mycoplasma contamination usng MycoAlert Mycoplasma Detection kit (Lonza, LT-07-318).
Commonly misidentified lines (See ICLAC register)	No ICLAC cell lines were used in this study
