# Supplementary figures and images for: Structural basis for mTORC1 regulation by the CASTOR1–GATOR2 complex
Source: Nat Struct Mol Biol. 2025 Jul 25;32(10):1980–8. doi: 10.1038/s41594-025-01635-0 (PMC12477443; doi:10.1038/s41594-025-01635-0)

Figure 3g

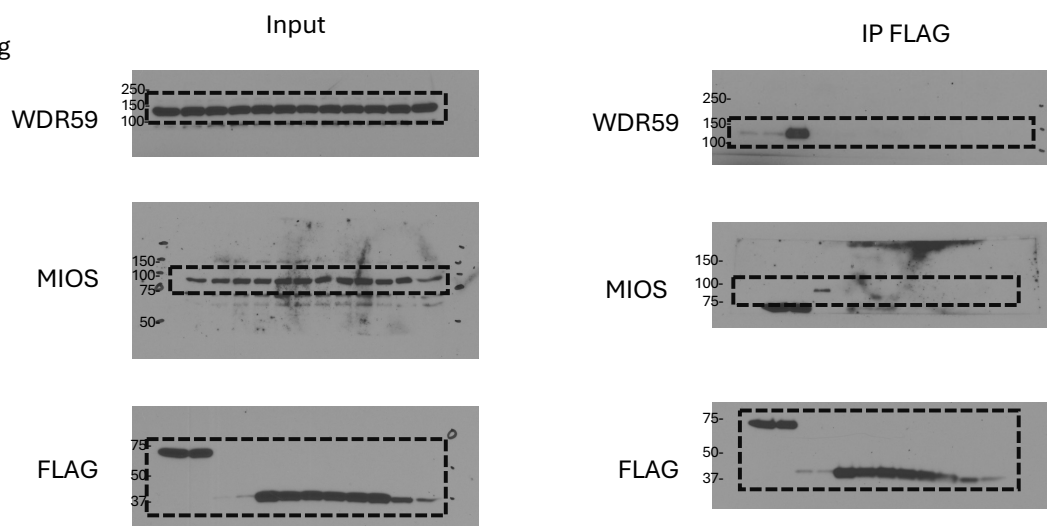

Figure 3h

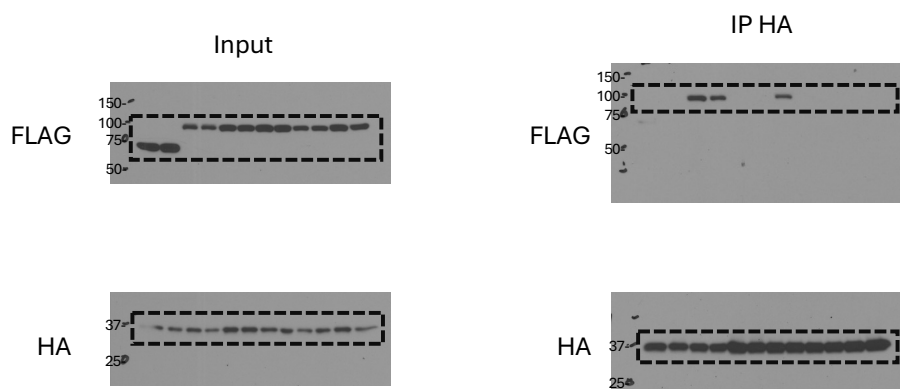

Figure 3i

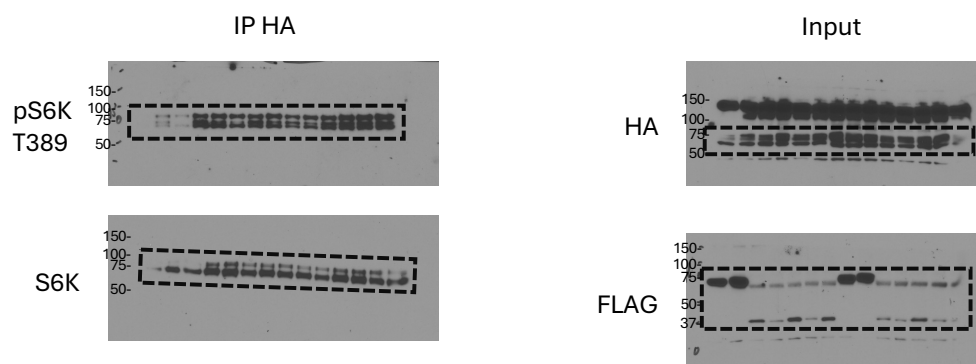

Supplement: Supplementary file 4 — Unprocessed western blots. [file 41594_2025_1635_MOESM4_ESM.pdf]

Figure 4d

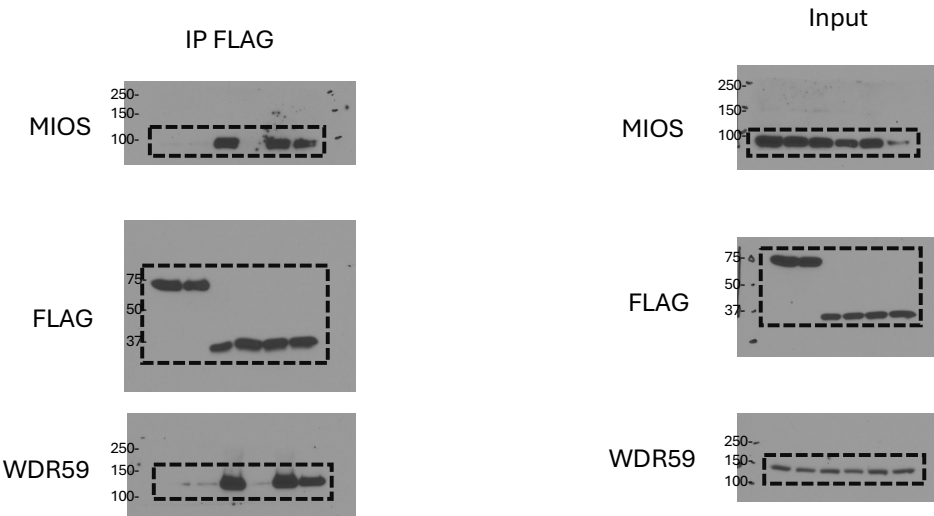

Figure 4e

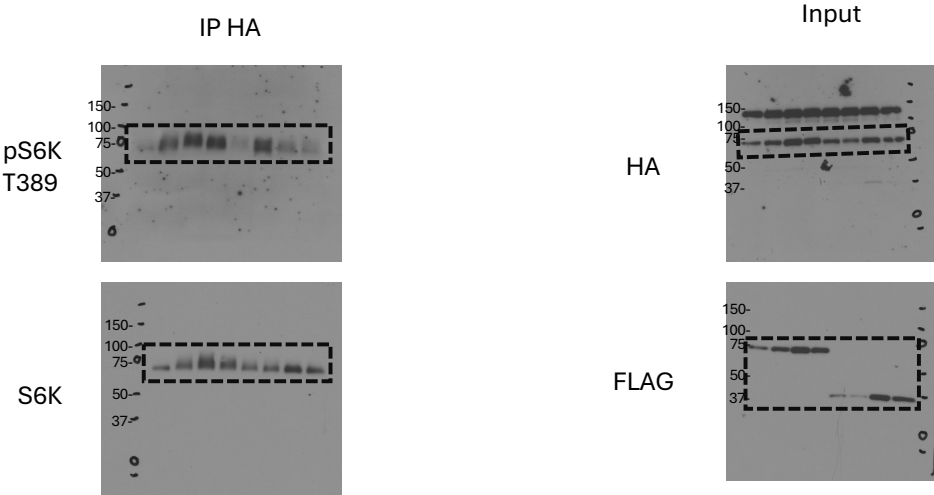

Supplement: Supplementary file 5 — Unprocessed western blots. [file 41594_2025_1635_MOESM5_ESM.pdf]

Supplemental Figure 9f

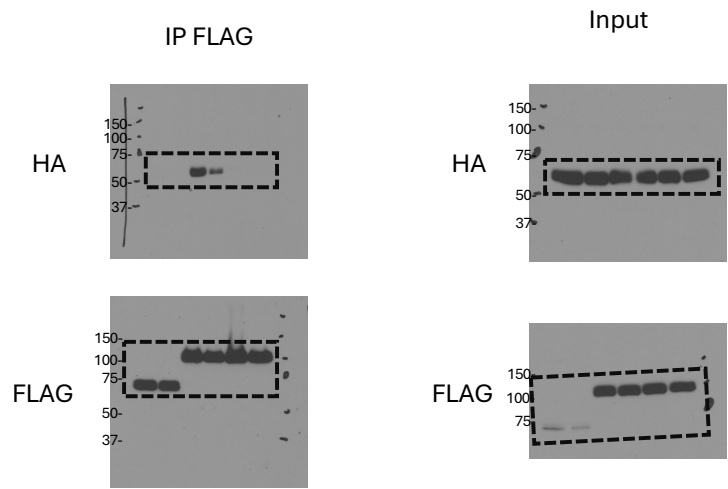

Supplement: Supplementary file 6 — Unprocessed western blots. [file 41594_2025_1635_MOESM6_ESM.pdf]
